# Reweighting of Binaural Localization Cues in Bilateral Cochlear-Implant Listeners

**DOI:** 10.1007/s10162-021-00821-3

**Published:** 2021-11-23

**Authors:** Maike Klingel, Bernhard Laback

**Affiliations:** 1 Department of Cognition, Emotion, and Methods in Psychology, Faculty of Psychology, University of Vienna, Vienna, Austria; 2 Acoustics Research Institute, Austrian Academy of Sciences, Vienna, Austria

**Keywords:** Interaural time difference, Interaural level difference, Plasticity, Training, Thresholds

## Abstract

Normal-hearing (NH) listeners rely on two binaural cues, the interaural time (ITD) and level difference (ILD), for azimuthal sound localization. Cochlear-implant (CI) listeners, however, rely almost entirely on ILDs. One reason is that present-day clinical CI stimulation strategies do not convey salient ITD cues. But even when presenting ITDs under optimal conditions using a research interface, ITD sensitivity is lower in CI compared to NH listeners. Since it has recently been shown that NH listeners change their ITD/ILD weighting when only one of the cues is consistent with visual information, such reweighting might add to CI listeners’ low perceptual contribution of ITDs, given their daily exposure to reliable ILDs but unreliable ITDs. Six bilateral CI listeners completed a multi-day lateralization training visually reinforcing ITDs, flanked by a pre- and post-measurement of ITD/ILD weights without visual reinforcement. Using direct electric stimulation, we presented 100- and 300-pps pulse trains at a single interaurally place-matched electrode pair, conveying ITDs and ILDs in various spatially consistent and inconsistent combinations. The listeners’ task was to lateralize the stimuli in a virtual environment. Additionally, ITD and ILD thresholds were measured before and after training. For 100-pps stimuli, the lateralization training increased the contribution of ITDs slightly, but significantly. Thresholds were neither affected by the training nor correlated with weights. For 300-pps stimuli, ITD weights were lower and ITD thresholds larger, but there was no effect of training. On average across test sessions, adding azimuth-dependent ITDs to stimuli containing ILDs increased the extent of lateralization for both 100- and 300-pps stimuli. The results suggest that low-rate ITD cues, robustly encoded with future CI systems, may be better exploitable for sound localization after increasing their perceptual weight via training.

## INTRODUCTION

Binaural hearing allows listeners to localize sound sources and facilitates understanding speech in noise. Consequently, bilateral cochlear implantation is becoming the standard treatment for bilateral severe to profound deafness and has proven to be advantageous over unilateral cochlear-implant (CI) use. However, even when using both implants, CI listeners generally perform worse than normal-hearing (NH) listeners in the above-mentioned tasks (Kan and Litovsky 2015).

NH listeners rely on two binaural cues for azimuthal sound localization (i.e., in the horizontal dimension, referred to as lateralization), the interaural time difference (ITD) and the interaural level difference (ILD). ITD cues are conveyed via the carrier signal (i.e., the temporal fine structure) at low frequencies and via the temporal envelope at high carrier frequencies. ILDs, on the other hand, are physically negligible at low frequencies but provide strong lateralization cues at high frequencies (e.g., Macpherson and Middlebrooks 2002). Consequently, NH listeners weight ITD and ILD cues mainly based on the frequency content of the sound (i.e., the cues contribute to different amounts to the azimuthal percept) with ILDs dominating for high-frequency stimuli and ITDs dominating for low-frequency and wideband stimuli (Macpherson and Middlebrooks 2002). In contrast, bilateral CI recipients rely almost entirely on ILDs with little to no contribution of ITDs (Grantham et al. 2007; Seeber and Fastl 2008).

The reasons for the low contribution of ITD cues to lateralization of CI listeners are manifold. Technical factors include that present-day envelope-based stimulation strategies do not convey pulse-ITDs (i.e., ITD cues encoded via the pulse timing), and that envelope-ITD cues, which are conveyed by such strategies, are not sufficiently salient for important everyday-life stimuli such as speech because their envelope shape is typically too shallow (Laback et al. 2004,  2011). Furthermore, these envelope-based stimulation strategies typically use high-rate pulse carriers whereas CI listeners’ sensitivity (both in terms of thresholds and extent of lateralization) to ITD cues, conveyed via the pulse timing using a research interface that bypasses the clinical CI system, deteriorates for pulse rates above 100–200 pps (Laback et al. 2015). Therefore, even if ITD information was conveyed via the pulse timing, it would not be perceivable and CI listeners who use envelope-based stimulation strategies thus do not have access to reliable and salient ITD cues in their everyday lives.

Consequently, strategies aiming to better transmit ITD cues are being developed. One approach is to encode acoustic fine-structure cues via the pulse timing using low pulse rates [e.g., FSP (Hochmair et al. 2006), PDT (van Hoesel et al. 2008), PP (Williges et al. 2018)]. However, even though such a “fine-structure” processing strategy (FS4) improved ITD detection thresholds for pure-tone stimuli of a subset of CI listeners (Zirn et al. 2016), these benefits do not or only slightly translate to real-life situations such as sound localization (van Hoesel et al. 2008; Dillon et al. 2016) or speech understanding in noise (Dillon et al. 2016; Magnusson 2011; de Bodt and van de Heyning 2014; Riss et al. 2011; van Hoesel et al. 2008; Zirn et al. 2016).

In addition, perceptual factors play a role in the low contribution of ITDs in electric hearing. Even when ITDs are presented under the most favorable conditions (i.e., via the pulse timing and at a single interaurally place-matched electrode pair, directly stimulated via a research interface using the most sensitive pulse rate), CI listeners’ ITD sensitivity is lower and much more variable across listeners compared to that of NH listeners (Laback et al. 2007; Majdak et al. 2006; van Hoesel 2007; Thakkar et al. 2020). Several explanations have been proposed for this perceptual deficit in electric hearing (see Laback et al. 2015, for a review). For instance, deprivation of binaural experience due to long periods before or between implantation on both ears results in cortical anomalies (Gordon et al. 2010) as well as degraded neural ITD coding (Chung et al. 2019; Hancock et al. 2010). Accordingly, Thakkar et al. (2020) reported that ITD sensitivity is related to the duration of bilateral hearing impairment and to CI experience. Another factor may be a mismatch between stimulation rate and membrane and synaptic parameters of binaural cells, which may explain the decline in sensitivity with increasing rate (Chung et al. 2015). This rate limitation is not only observed in discrimination thresholds, but also affects lateralization ranges (i.e., how far stimuli are lateralized to the sides; Anderson et al. 2019). Interestingly, the decline in pulse-ITD sensitivity with increasing rate (for unmodulated pulse trains) matches the decline in envelope-ITD sensitivity with increasing modulation rate observed in acoustic hearing (e.g., Bernstein and Trahiotis 2002; van Hoesel 2007; Laback et al. 2007; Stecker and Brown 2010). Although the auditory nerve can follow temporal information at high electric stimulation rates (e.g., Dynes and Delgutte 1992), this suggests that the neural pathway that encodes high-rate carrier ITDs in normal hearing is not accessed in electric stimulation. Instead, pulse-ITDs appear to activate the envelope-ITD pathway.

Here, we investigate another mechanism potentially contributing to the low perceptual impact of optimally conveyed pulse-ITD cues in electric hearing. It is conceivable that the lack of reliable and salient pulse-ITD cues CI listeners experience in their everyday lives leads to reweighting of the binaural cues, i.e., a change in the relative contribution of the cues to sound lateralization: CI listeners might learn over time to increase the perceptual weighting of ILDs which correctly indicate the location of many sound sources in daily life, and decrease the perceptual weighting of pulse-ITDs which arise at random or are not perceived at all. This seems plausible given that NH listeners reweight binaural localization cues based on the reliability of each cue: They showed increased weighting for ILDs and correspondingly reduced weighting for ITDs after task-irrelevant auditory stimuli containing stable ILD and random ITD cues were presented during a visual oddball task (Kumpik et al. 2019). Furthermore, a recent study in our lab showed that the relative weight of either ITDs or ILDs can be increased in NH listeners, depending on which cue is reinforced during a lateralization training in a virtual audio-visual environment by presenting visual cues spatially consistent with one binaural cue while varying the other cue (Klingel et al. 2021). We are now interested in whether an increased weighting for ILDs in CI listeners can be reversed by presenting reliable pulse-ITDs (referred to as ITDs in the following) and varying ILDs.

Therefore, this study investigates whether the paradigm used by Klingel et al. (2021) can induce an increase in ITD weighting in CI listeners. We were further interested in how the perceptual weight given to each binaural cue relates to binaural-cue thresholds and whether binaural-cue reweighting will also be reflected in a change in binaural-cue thresholds (i.e., whether binaural-cue weighting is simply a reflection of the sensitivity to each binaural cue or whether it is a supra-threshold mechanism). We tested two pulse rates. The 100-pps condition explored reweighting for stimuli that provide the highest ITD sensitivity. The 300-pps condition (representing any carrier rate that is too high to permit pulse-ITD sensitivity) investigated whether the rate limitation is connected to reweighting. Specifically, if reweighting contributes to the rate limitation, given that CI listeners are mostly exposed to high-rate carriers with their clinical devices, we may see reweighting at 300 pps. However, if the rate limitation results from a processing deficit independent of reweighting, no effect of training is expected at that rate. Finally, unrelated to binaural-cue reweighting, we addressed the practically relevant question if the combined presentation of salient ITDs and ILDs is beneficial for lateralization. Specifically, we tested whether the additional presentation of nonzero ITDs significantly contributes to the extent of lateralization beyond lateralization based on ILDs alone.

## METHODS

### Participants

Six CI listeners, bilaterally implanted with MED-EL Corp. devices (Innsbruck, Austria), completed the experiment. Two additional listeners participated in the study but were excluded: One listener experienced dizziness induced by the head-mounted display and dropped out of the study. The other listener had difficulties lateralizing the stimuli without visual reinforcement leading to most stimuli being perceived at either − 90° or + 90° azimuth. The listener and device information are summarized in Table 1. All listeners gave informed consent and received monetary compensation for their participation. The research protocol was submitted to the Acoustics Research Institute’s ethics committee for consideration, comments, guidance, and approval. After taking ethical issues, local laws, and regulations into account, the ethical committee approved the protocol.

**Table 1:** Listener data. The italicized listeners were excluded from the analyses

Listener	Implant L	Implant R	Age at testing	Etiology	Age at onset of deafness	Age at implantation (L/R)	Electrode tested (L/R)	ITDs doubled (100/300 pps)	Pulse rate trained first
CI3	C40 +	C40 +	35	Meningitis	20	20/20	11/10	No/No	300
*CI17*	*Synchrony*	*Synchrony*	*72*	*Idiopathic*	*41*	*69/58*	*8/8*	*Yes/Not tested*	*100*
CI62	C40 +	C40 +	19	Connexin 26	0	2/0	11/11	No/No	300
CI66	Synchrony	Concerto	55	Progressive	Childhood	37/46	8/6	No/Yes	300
CI74	Concerto	Sonata	49	Progressive	Adulthood	43/42	8/9	No/No	300
*CI77*	*Sonata*	*Sonata*	*66*	*Sudden hearing loss*	*33 (L)/57 (R)*	*58/57*	*8/6*	*No/Yes*	*100*
CI100	Implant L	C40 +	22	Unknown	Childhood	8/2	9/9	Yes/Yes	100
CI117	C40 +	C40 +	38	Etiology	Early adulthood	37/22	8/9	Yes/Yes	100

### Apparatus and Stimuli

**Fig. 1:**
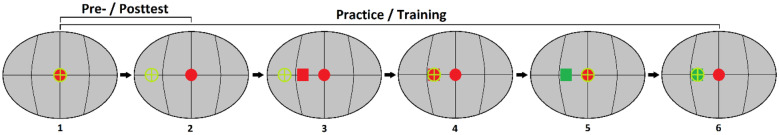
Time course of a trial during the binaural-cue weight measurement (panels 1–2) and the practice session as well as the lateralization training (panels 1–6). (1) Listeners oriented towards the reference position (indicated by a red sphere) and pressed a button to elicit the sound presentation. (2) Listeners turned their head (guiding a green crosshair) to the perceived azimuth and pressed the button (in this example, they turned their head to the left). (3) Visual reinforcement (a rotating red cube) appeared at the ITD azimuth. (4) The ITD azimuth was confirmed via a head-turn to the visual reinforcement and a button-press. (5) The visual reinforcement turned green, listeners returned to the reference position, and elicited the second sound presentation (while the visual reinforcement was still visible) with another button-press. (6) Listeners confirmed the ITD azimuth again via another head-turn and button-press. In steps (4) and (6), the button-press was accepted only if the head orientation (green crosshair) was within ± 5° of the ITD azimuth

For the binaural-cue weight measurements as well as the training, a lateralization task in a virtual audio-visual environment was used. The virtual environment served to mediate the lateralization process and to present visual reinforcement during the lateralization training. Visual stimuli were presented binocularly via a head-mounted display (Oculus Rift DK1). The visual environment (Fig. 1) consisted of a reference position straight ahead, a crosshair in the direction the head is oriented, a single horizontal line at eye level, and vertical lines every 15° in azimuth for guidance. A rotating cube was used as visual reinforcement. Listeners were seated on a desk chair that was allowed to rotate. Their head position and orientation were tracked with a head-mounted tracking sensor (Flock of Birds, Ascension) and the visual environment was rendered accordingly in real time (with a latency between head movements and the updated visual information of less than 37.3 ms). The rendering of the visual environment using Pure Data (with GEM, IEM, Graz) was based on the left/right rotation information from the tracking sensor while the up/down information was ignored to force listeners to respond only in the horizontal plane.

Auditory stimuli were generated on a personal computer and were directly presented to the two CIs via a research interface (RIB2) developed at the Institute of Ion Physics and Applied Physics, Leopold-Franzens University of Innsbruck, Austria, allowing precisely controlled interaurally coordinated stimulation. Listeners were thus isolated from any audio-visual signals besides the experimental stimuli during the experiment, while they used their clinical devices during breaks and between sessions. Prior to the main experiment, the threshold (THR), comfortable level (CL), and maximum comfortable level (MCL) were determined manually using a continuous scale from “not audible” to “uncomfortably loud”. For stimuli with zero ILD, the CL measured at each ear was used (for stimuli including ILDs, see below). All stimulation pulses were symmetric-, biphasic-, and cathodic-phase leading. The duration of a phase was 26.7 μs and there was no inter-phase gap. If sufficient loudness could not be reached, the phase duration was increased to 40 μs.

Auditory stimuli in the main experiment were unmodulated electric pulse trains with a pulse rate of either 100 or 300 pps and a duration of 500 ms, presented at a single interaurally place-matched electrode pair (see “5” for details and Table 1 for chosen electrodes). Various combinations of ITDs and ILDs were imposed on these stimuli. A range of ILDs was determined individually for each listener during the parameter determination (see “5” for details), with the goal to elicit a set of perceived azimuths (referred to as ILD azimuths) equally spaced between − 69° (left) and + 69° (right). This range is analogous to Klingel et al. (2021). For ITDs, we did not determine listener-specific ITDs matching that azimuth range, because it was foreseeable from pilot tests and other studies (e.g., Anderson et al. 2019) that listeners would never lateralize across such a wide range of azimuths. Thus, we accepted that ILDs would dominate the lateralization percept in the pretest, while leaving room for increasing the contribution of ITDs. Instead, naturally occurring ITDs were used that ranged from − 654 to + 654 μs, corresponding to an azimuth range from − 69° to + 69° (referred to as ITD azimuths), as estimated by Xie (2013) using broadband cross-correlation of the head-related impulse responses of the KEMAR head with DB-61 small pinna at a source distance of 1.4 m. If the listener’s left/right discrimination threshold for ITDs was larger than two times the largest ITD used (i.e., 1308 μs), all ITDs were doubled^1^ to increase the likelihood that ITDs provided useful lateralization cues (see Table 1). For CI74, ITDs were not doubled at 300 pps, even though that listener’s threshold exceeded 1308 μs. However, we believe this does not change the overall interpretation of the results, since CI74’s 300-pps threshold exceeded 3200 μs and doubling the ITDs would therefore have been unlikely to affect the results. ILDs are reported in percent of the dynamic range (%DR) between the threshold (THR), comfortable level (CL), and maximum comfortable level (MCL) of each listener (Eq. 1).

$$\mathrm{If}\;\mathrm{ILD}(\%\mathrm{DR})<0:\;{\mathrm{Amp}}_{\mathrm L}={\mathrm{CL}}_{\mathrm L}+\frac{-\mathrm{ILD}\ast\left({\mathrm{MCL}}_{\mathrm L}-{\mathrm{CL}}_{\mathrm L}\right)}{200};{\mathrm{Amp}}_{\mathrm R}={\mathrm{CL}}_{\mathrm R}-\frac{-\mathrm{ILD}\ast({\mathrm{CL}}_{\mathrm R}-{\mathrm{THR}}_{\mathrm R})}{100}$$

1 $$\mathrm{If}\;\mathrm{ILD}(\%\mathrm{DR})>0:{\mathrm{Amp}}_{\mathrm L}={\mathrm{CL}}_{\mathrm L}-\frac{\mathrm{ILD}\ast\left({\mathrm{CL}}_{\mathrm L}-{\mathrm{THR}}_{\mathrm L}\right)}{100};{\mathrm{Amp}}_{\mathrm R}={\mathrm{CL}}_{\mathrm R}+\frac{\mathrm{ILD}\ast({\mathrm{MCL}}_{\mathrm R}-{\mathrm{CL}}_{\mathrm R})}{200}$$

This ILD specification aimed at providing roughly constant loudness across a wide range of ILDs, following earlier research (Best et al. 2011).

**Fig. 2:**
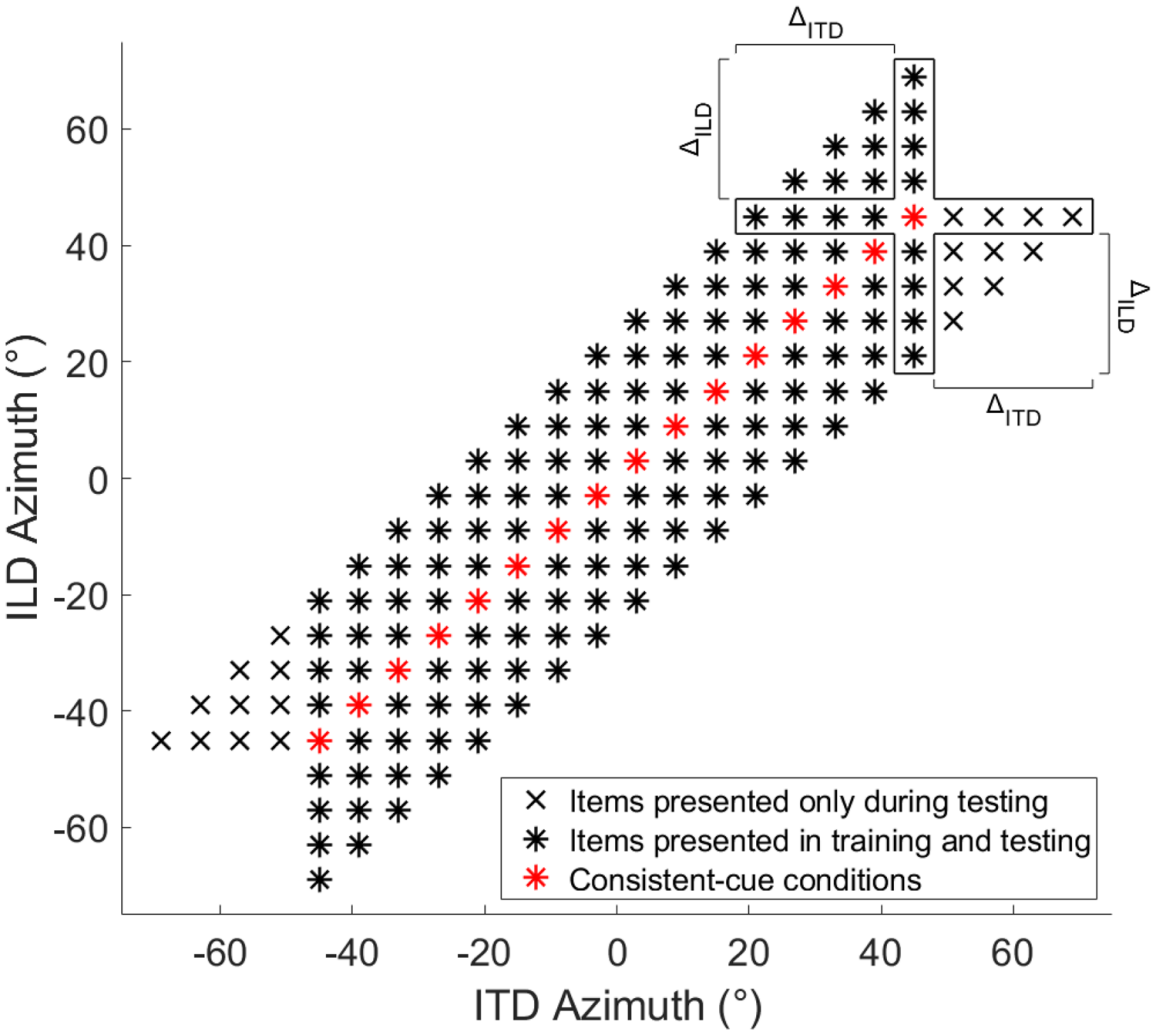
ITD/ILD combinations presented in this study. During lateralization training, ILD azimuths were uniformly distributed around each ITD azimuth (columns of asterisks). During the lateralization tests used for estimating binaural-cue weights, ITD/ILD combinations marked with an “x” were additionally presented to also ensure a uniform distribution of ITD azimuths around each ILD azimuth, which was needed for the binaural-cue weight estimation (the frame indicates items that were used to estimate the ITD weight at an example azimuth of 45°)

ITDs and ILDs were then combined into “consistent-cue” and “inconsistent-cue” conditions. In consistent-cue conditions, the ITD and ILD cue of the auditory stimulus corresponded to the same azimuth (red asterisks along the diagonal in Fig. 2), while they corresponded to disparate azimuths in inconsistent-cue conditions. Recall that ILD azimuths are psychophysical estimates based on each listener’s perception while ITD azimuths are based on acoustical measurements in a dummy head. Therefore, consistent-cue stimuli are only nominally consistent and might not reflect a perceptually consistent binaural-cue combination. To address this, listeners could familiarize themselves with the consistent-cue stimuli during a practice session with each pulse rate (see below). Furthermore, since the parameters were held constant throughout the experiment, potential mismatches should not influence the comparisons across test phases. Cue disparities (i.e., the difference between the ITD and ILD azimuth of a stimulus) were restricted to a maximal value of 24°, to avoid the perception of split images that can occur in case of large cue disparities (Gaik 1993). In the lateralization tests, ITD and ILD cues were uniformly distributed ± 24° around each azimuth on the diagonal of Fig. 2, in both the ITD and ILD dimensions (all symbols in Fig. 2 in rows and columns, respectively). In the lateralization training, ITD azimuths were visually reinforced, and their range was restricted to ± 45° (all asterisks in Fig. 2). The restricted ITD azimuth range was chosen to ensure that the visual reinforcement was always visible when facing the reference position (i.e., ± 45° was the field of view of the head-mounted display), encouraging multisensory bottom-up processes during simultaneous presentation of the auditory stimulus and visual reinforcement. Moreover, by symmetrically varying the ILD azimuth around each ITD azimuth [resulting in a larger range of ILD azimuths (± 69°) than ITD azimuths (± 45°)], the ITD was the more stable cue which might further encourage reweighting (Dahmen et al. 2010).

For the threshold measurements, listeners were seated in front of the screen of a personal computer and gave left/right responses with a gamepad. The auditory stimuli, unmodulated 100- or 300-pps pulse trains, were again directly presented to the two CIs via the RIB2.

### Procedure

**Fig. 3:**
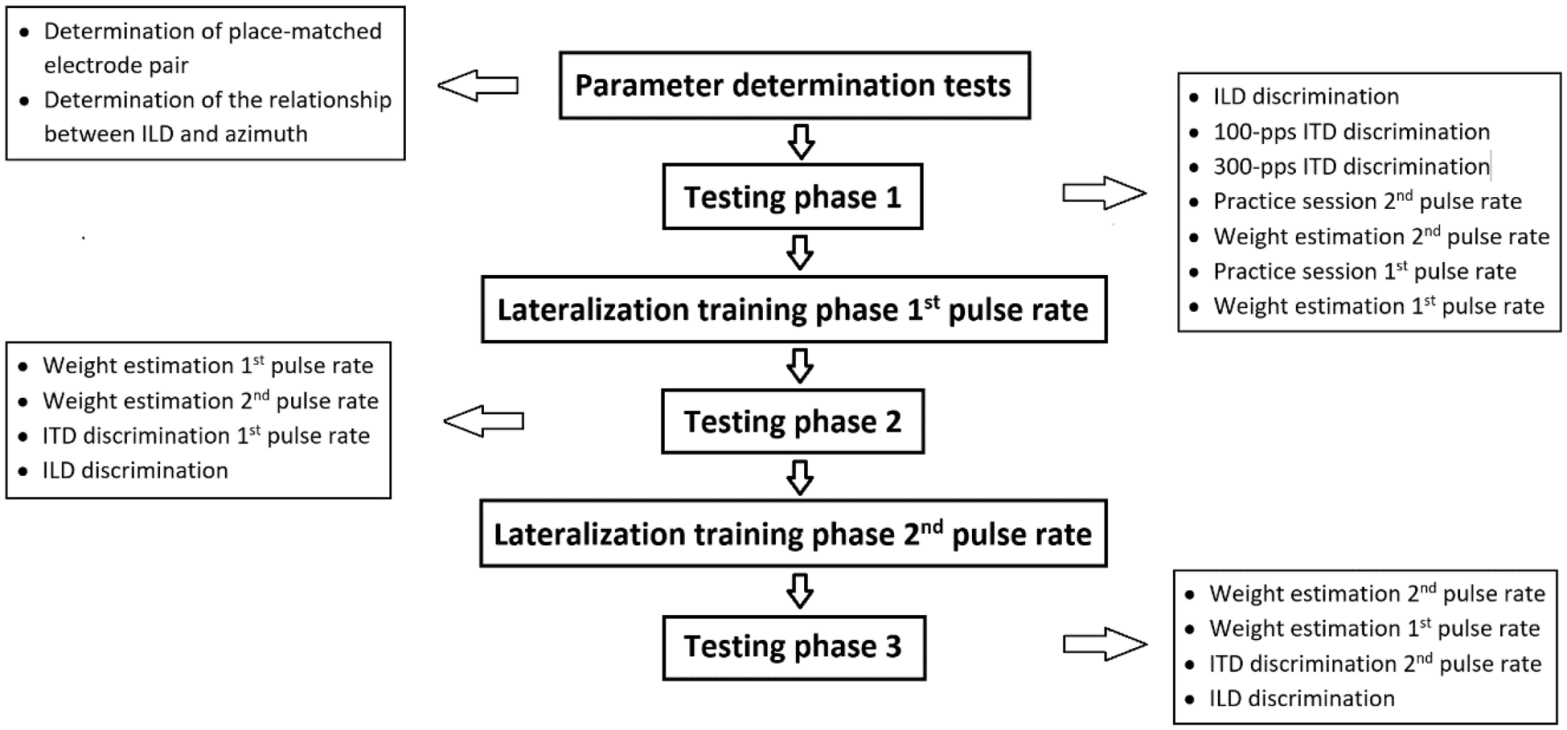
Experimental structure. The experimental phases, completed from top to bottom, are shown in the center. The boxes on the sides show the tests completed during parameter determination and during each testing phase, also sorted top to bottom

The general structure of the experiment is shown in Fig. 3. At the beginning, several tests were performed to determine the parameters for the main experiment. In the testing phases [before (1), in-between (2), or after (3) the two lateralization training phases reinforcing ITD cues], binaural-cue weights (via a lateralization task) as well as binaural-cue sensitivity (via a discrimination task) were measured. Which of the two pulse rates was trained first was counter-balanced across listeners. However, the drop-out of two listeners resulted in 4 of the included listeners starting with 300-pps training while 2 listeners started with 100-pps training. The data collection per listener took 4–5 days to complete.

#### Parameter Determination

We determined a place-matched binaural electrode pair using a well-established procedure (e.g., Majdak et al. 2006, or Laback et al. 2007) using unmodulated 1515-pps pulse trains to reduce the potentially confounding effect of rate pitch at lower rates. That procedure involved pitch magnitude estimation of loudness-matched stimulation electrodes at both ears followed by pitch discrimination of pitch-matched pair candidates. An electrode pair at the basal region that elicited pitch discrimination at chance level was then selected for the rest of the study. We chose a basal electrode pair to provide the best conditions for ITD sensitivity (see Laback et al. 2015). Furthermore, assuming that the pulse rate in electric stimulation is perceptually analogous to the envelope rate in acoustic hearing (Stecker and Brown 2010), basal electrodes correspond to the cochlear place where envelope cues are represented in normal hearing. If a place-matched electrode pair had already been determined for a listener in a previous study at the authors’ lab using the same methodology (as for CI3, CI62, CI100, and CI74), that pair was also used in the present study. We then determined THR, CL, and MCL separately for 100 and 300 pps at the place-matched electrode pair members.

**Fig. 4:**
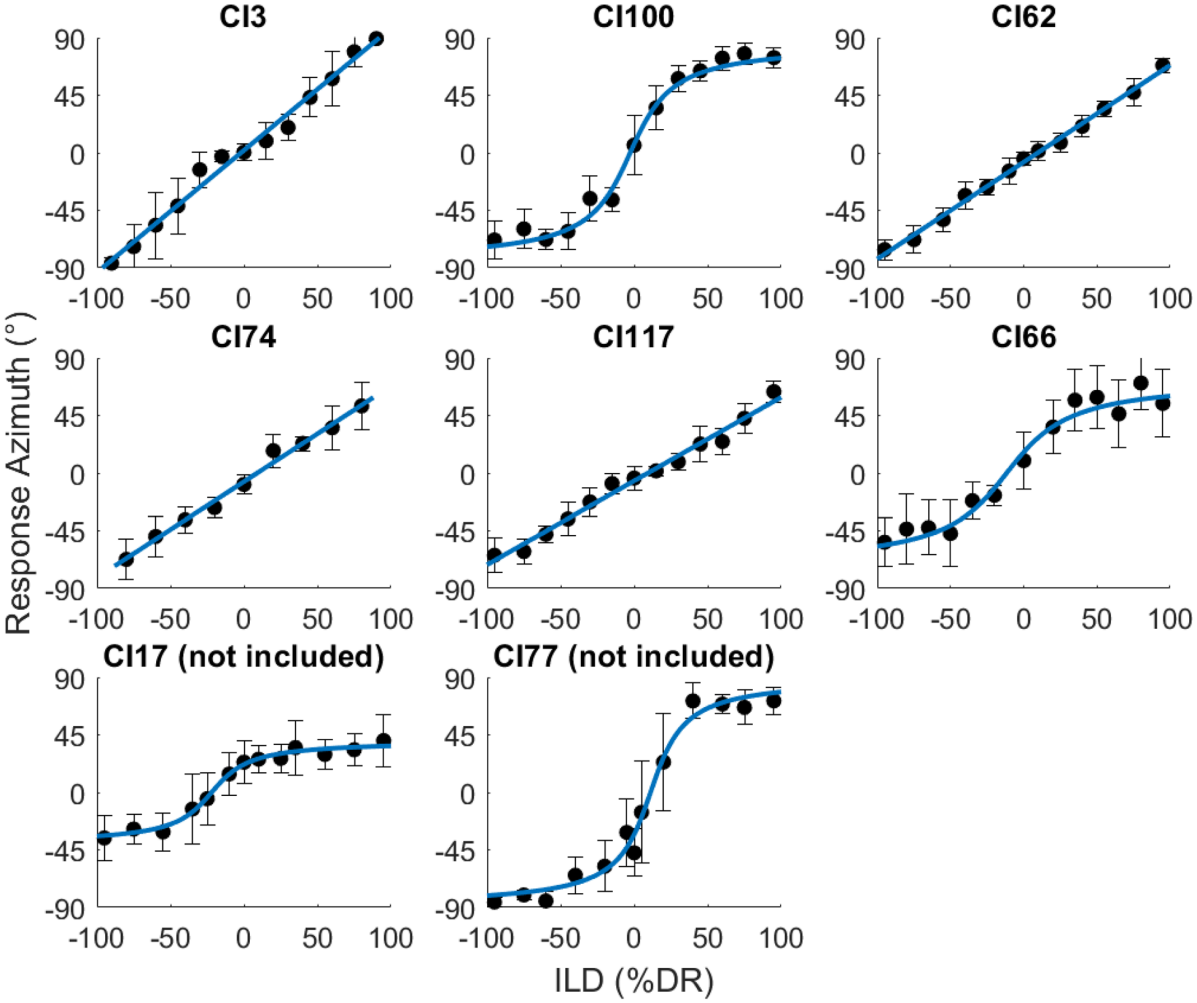
Lateralization data used to determine the relationship between ILDs and perceived azimuths. Error bars show standard errors of the mean. The blue lines show the fitted functions. Linear fits were used for CI3, CI62, CI74, and CI117, inverse tangent fits were used for CI100, CI66, CI17, and CI77. CI17 and CI77 were excluded from the study

**Fig. 5:**
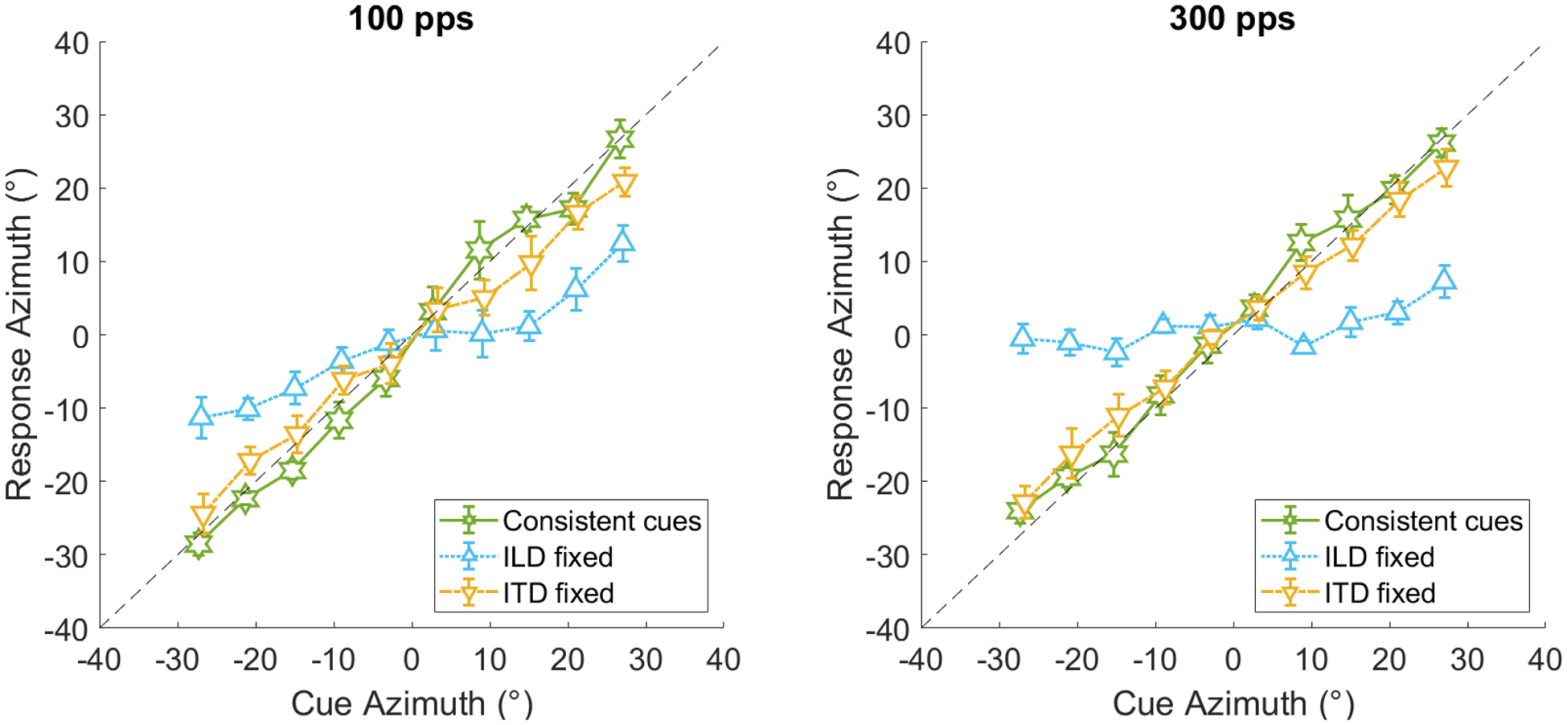
Mean response azimuths for varying ITDs (ILDs fixed at ± 3°), varying ILDs (ITDs fixed at ± 3°) and for consistent cues after averaging across all 3 test phases. Results for the 100-pps condition are shown on the left and results for the 300-pps condition are shown on the right. Error bars show the standard error of the mean. Note that varying ITDs and ILDs correspond to the parameters Δ_ITD_ and Δ_ILD_, respectively, in the regression analysis (Eq. 2)

To determine the relationship between ILD and perceived azimuth, we presented 6 left- and 6 right-leading ILDs (10 repetitions each) and asked the listeners to lateralize the stimuli in the virtual environment (see steps 1 and 2 in Fig. 1). On each trial, participants listened to the auditory stimulus while facing the reference position and then indicated the perceived azimuth via head turn and button press. After they returned to the reference position, the next auditory stimulus was presented. We then averaged the response azimuths for each ILD and fitted either a linear function or an inverse tangent function, depending on which fitted the data better as indicated by a higher R^2^ (see Fig. 4). Finally, ILDs were read out from the fitted function to obtain ILDs corresponding to the azimuths used in the main experiment (thus, defining the ILD azimuths). Due to limited testing time and because we did not expect the functions to differ between pulse rates, only 100-pps stimuli were used for fitting ILD values to azimuths. The assumption of comparable lateralization functions for 100- and 300-pps pulse trains was confirmed post hoc (see yellow downward pointing triangles in Fig. 5).^2^

#### Testing Phase

The sequence of measurements performed during each testing phase is shown in Fig. 3. In all testing phases, we measured binaural-cue weights for both pulse rates with a lateralization task as well as binaural cue thresholds with a discrimination task. In testing phase 1 only, listeners additionally performed a practice session including visual reinforcement (see description of the training task below) to get accustomed to the lateralization training task. In the practice session, only consistent-cue combinations (red asterisks in Fig. 2) were presented, 4 repetitions per azimuth (i.e., 64 trials total). This was done separately with 100- and 300-pps stimuli right before the respective lateralization test.

Binaural-cue weights were measured with a lateralization task in the virtual audio-visual environment without visual reinforcement. Namely, by obtaining lateralization responses for stimuli containing spatially inconsistent ITD and ILD, it can be inferred how much each cue contributes to the azimuthal percept by comparing the response azimuth to the respective binaural-cue azimuths (Macpherson and Middlebrooks 2002; see “10” below). On each trial, the auditory stimulus was presented while listeners faced the reference position and then indicated the perceived azimuth via head turn and button press. When they returned to the reference position, the next item was presented (steps 1 and 2 in Fig. 1). After each 124 trials, listeners took a short break. Each ITD/ILD combination (i.e., all data points shown in Fig. 2) was presented 3 times, resulting in 492 trials in total. In addition, a centered item was presented at the beginning as well as after each break to help listeners build up their audio-visual reference frame. This measurement was done in separate blocks for the 100- and 300-pps stimuli.

For measuring ITD and ILD thresholds, we used a constant-stimuli, cued left/right discrimination paradigm. The first interval of each trial consisted of a centered stimulus (zero ITD and zero ILD; note that zero ILD refers to an ILD causing a centered image). The second interval consisted of the same stimulus with, depending on which cue was tested, either a nonzero ITD or a nonzero ILD while the other was left at zero. The listeners had to indicate on which side (left or right) the second stimulus was perceived compared to the first stimulus by pressing the corresponding button on a gamepad. They received feedback (correct/incorrect) after each response. During ITD threshold estimation, we tested ITDs of ± 100, ± 200, ± 400, ± 800, and ± 1600 μs. If it was known from previous tests that the 100-pps threshold of a listener was outside of this range, the range of ITDs tested was adjusted accordingly (smallest ITD tested: 25 μs). ITD thresholds were measured separately for 100 and 300 pps in testing phase 1 and after the training phase with the respective pulse rate. During ILD threshold estimation, ILDs were chosen based on the listener’s ILD lateralization results during parameter determination. ILD thresholds were measured using 100-pps stimuli during all three testing phases. For both ITD and ILD threshold estimations, 100 repetitions were tested per cue value.

#### Lateralization Training Phase

Listeners were trained with a lateralization task in the virtual audio-visual environment using visual reinforcement consistent with the ITD azimuth (Fig. 1). A training trial started with the presentation of the auditory stimulus while listeners faced the reference position. They indicated the perceived azimuth via head turn and button press, so far identical to the lateralization testing trials described above. It proceeded with visual reinforcement appearing at the ITD azimuth. Listeners were instructed to perform a corrective head turn from the response azimuth to the displayed azimuth and confirm it with a button press. When they returned to the reference position and pressed the button, the same auditory stimulus was presented again, now simultaneously with the visual reinforcement. Then, the displayed azimuth had to be confirmed again via another head turn and button press. Finally, listeners returned to the reference position to start a new trial. Thus, the training utilized two forms of visually guided auditory spatial calibration: (1) visual stimuli presented after the auditory stimuli, serving as top-down (i.e., involving cognition) feedback comparable to the “feedback” experiments of Shinn-Cunningham et al. (1998), and (2) simultaneously presented auditory and visual stimuli, encouraging multisensory bottom-up (i.e., stimulus-driven) processes equivalent to those evoked in the ventriloquism aftereffect paradigm (Recanzone 1998). After each 73 trials, listeners took a short break. One training block consisted of 146 trials, namely each ITD azimuth combined with each ILD azimuth ± 24° around that ITD azimuth (columns of asterisks in Fig. 2) plus a centered item at the beginning and after each break to help listeners to orient themselves. Listeners completed five training blocks per pulse rate.

### Analysis

Analogous to Klingel et al. (2021), we estimated binaural-cue weights individually for each listener based on a regression analysis motivated by the experimental design, which measured the effect of varying the ILD on the responses at each ITD azimuth and vice versa. A resulting ITD weight of 1 means that listeners always responded at the ITD azimuth, an ITD weight of 0 means listeners always responded at the ILD azimuth, and an ITD weight of 0.5 represents equal weighting of the two cues. The regression analysis was fitted separately for each azimuth α (between 3° and 45° with a 6° spacing between azimuths) after averaging repetitions and mirroring the data across the midline.^3^ The regression model equations are as follows:

$$R_{ITD}\left(\alpha,\Delta_{ITD}\right)=k_{ITD}\left(\alpha\right)\ast\Delta_{ITD}+Q_{ITD}\left(\alpha\right)$$

$$R_{ILD}\left(\alpha,\Delta_{ILD}\right)=k_{ILD}\left(\alpha\right)\ast\Delta_{ILD}+Q_{ILD}\left(\alpha\right)$$

$$w_{ITD}\left(\alpha\right)=\frac{atan\left(\frac{k_{ITD}\left(\alpha\right)}{k_{ILD}\left(\alpha\right)}\right)}{\frac{\pi}2}$$

2 $$Q\left(\alpha\right)=\frac{Q_{ILD}\left(\alpha\right)+Q_{ITD}\left(\alpha\right)}2$$

where R_ITD_ (R_ILD_^4^) is the listener’s mean response azimuth in a trial for which the ILD (ITD) corresponded to azimuth α and the ITD (ILD) corresponded to azimuth α + Δ_ITD_ (α + Δ_ILD_). The parameters k_ITD_ and k_ILD_ are the estimated linear regression slopes at azimuth α (determining the individual binaural-cue contributions), and Q is the estimated response azimuth for consistent-cue stimuli corresponding to azimuth α. Parameter k_ITD_ (k_ILD_) was estimated at each α by considering various azimuthal offsets (from − 24° to + 24° with 6° spacing) of the cue Δ_ITD_ (Δ_ILD_) while setting the offset of the other cue, Δ_ILD_ (Δ_ITD_), to zero. Thus, the model was fitted for each azimuth α, indicated by a red asterisk in Fig. 2, by considering only items of the row (for k_ITD_) and column (for k_ILD_) that include that red asterisk (indicated by the frame for an example azimuth of 45°). These estimates of k_ITD_ and k_ILD_, representing orthogonal vectors, were then combined to derive the ITD weight, w_ITD_ (note that w_ILD_ = 1 − w_ITD_). Finally, parameter Q was estimated as the average of the constants obtained in the regressions for ITD and ILD. To compare pre- and post-training weights across listeners, we averaged the estimated weights obtained before or after the lateralization training, depending on which pulse rate was trained first (i.e., for determining 100-pps weights, if the listener started with 100-pps training, lateralization test 1 constitutes the pretest and the estimated weights from lateralization tests 2 and 3 were averaged to constitute the posttest. If the listener started with 300-pps training, 100-pps weights from lateralization tests 1 and 2 were averaged to constitute the pretest and lateralization test 3 constitutes the posttest).

Binaural-cue thresholds at a performance level of 75% (i.e., halfway between chance and perfect performance) were determined with the MATLAB toolbox psignifit 4 (Schütt et al. 2015). ITD values were logarithmized using the natural logarithm before fitting the psychometric function. To determine ILD thresholds, the difference between the reference and target stimulus in current units (cu) was evaluated. As a conservative means to reflect that the listeners may not make judgments based on the difference between the reference and the target stimulus, but rather compare the target stimulus to previously presented targets, the obtained threshold values were doubled for both ITD and ILD thresholds (Hartmann and Rakerd 1989).

The data were analyzed using MATLAB R2018b (The MathWorks, Natick, MA). Statistical analyses were performed using SPSS Statistics 20 (IBM, Armonk, NY).

## RESULTS

### Binaural-Cue Weights

**Fig. 6:**
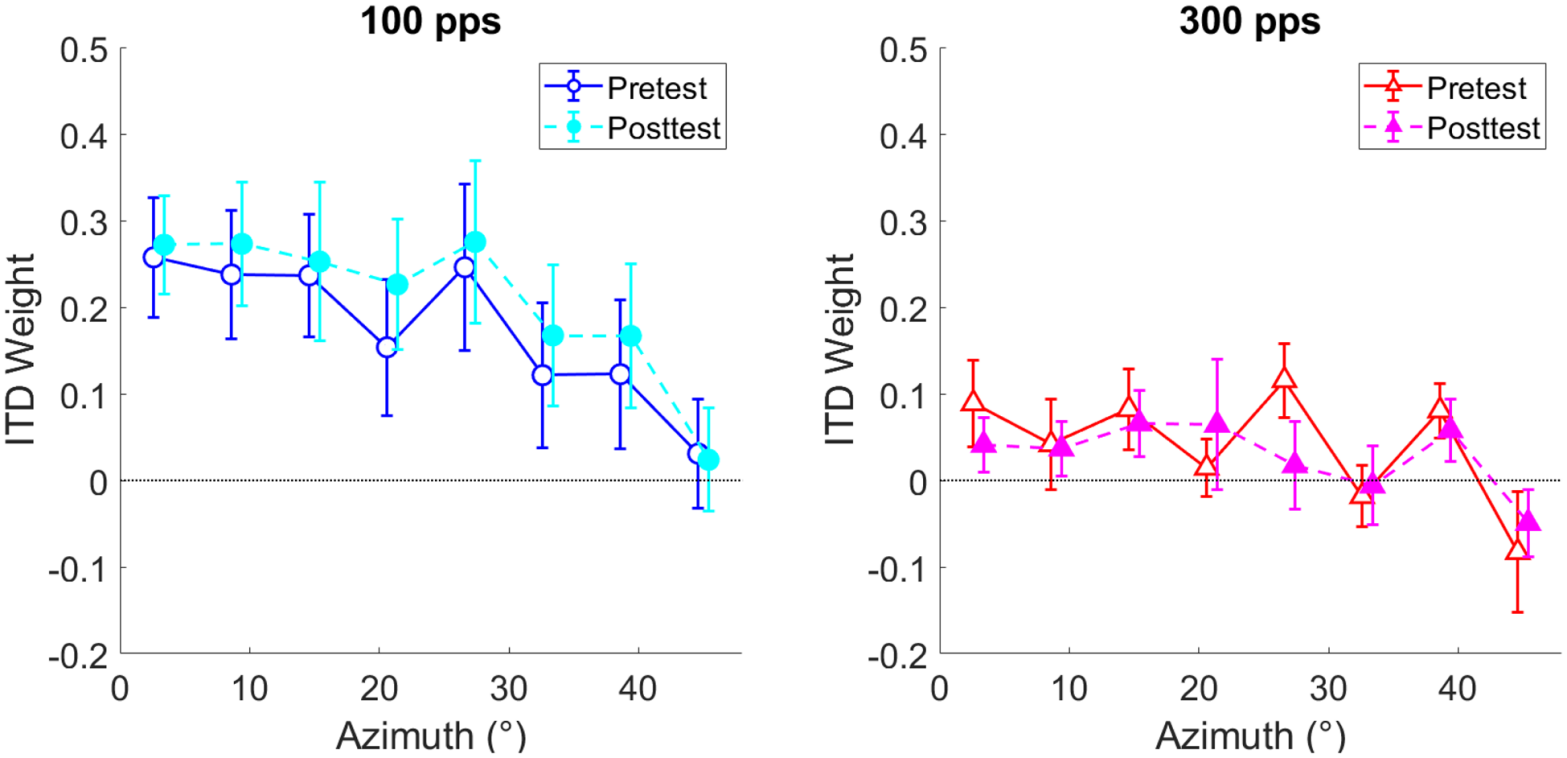
Mean ITD weights averaged across listeners as a function of azimuth for the 100-pps condition (left panel) and the 300-pps condition (right panel). Error bars show the standard error of the mean

Figure 6 shows the ITD weights averaged across listeners as a function of azimuth for the pretest (open symbols) and the posttest (filled symbols). Data for 100-pps stimuli are shown in the left panel and data for 300-pps stimuli are shown in the right panel. ITD weights were subjected to a 2 (100 vs. 300 pps) × 2 (pre- vs. posttest) × 8 (azimuth) repeated-measures (RM) ANOVA. The ANOVA yielded a significant main effect of pulse rate (F(1,5) = 10.26, p = 0.024, η_p_^2^ = 0.672) with larger ITD weights at 100 pps (M = 0.19, SD = 0.17) than 300 pps (M = 0.03, SD = 0.06), a significant main effect of azimuth (F(7,35) = 7.28, p < 0.000, η_p_^2^ = 0.593) with smaller ITD weights at more lateral azimuths, and a significant pulse rate × testing time interaction (F(1,5) = 8.76, p = 0.032, η_p_^2^ = 0.637). Post hoc pairwise comparisons showed that the pulse rate × testing time interaction was driven by a significant increase of ITD weights from pre- to posttest in the 100-pps condition (p = 0.046, Bonferroni-corrected), but no effect of testing time in the 300-pps condition (p = 0.443, Bonferroni-corrected). Taken together, these results suggest that listeners increased their ITD weighting for 100-pps but not for 300-pps stimuli as a function of testing time and that ITD weights were consistently lower for 300 pps and at lateral azimuths.

**Fig. 7:**
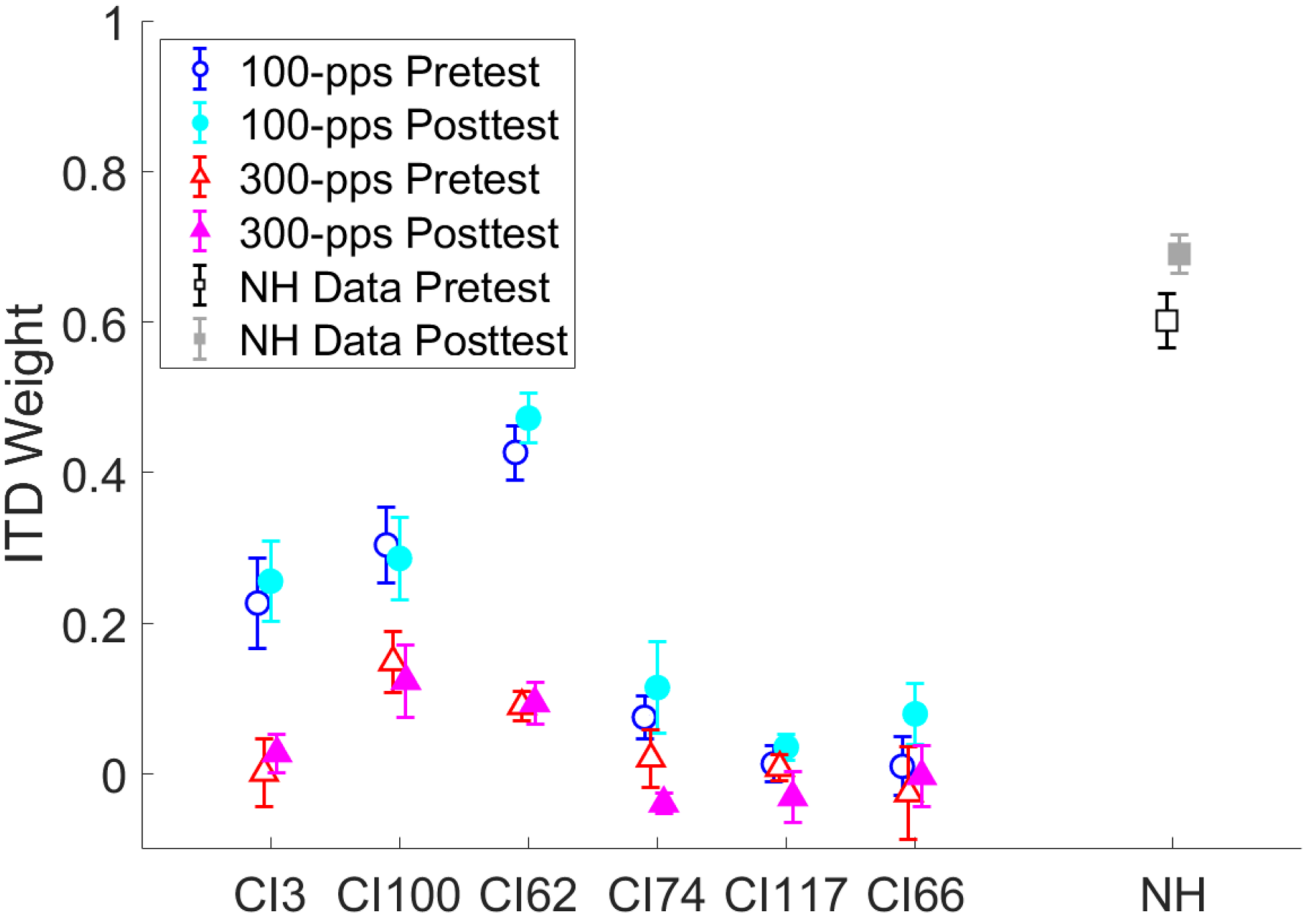
ITD weights averaged across azimuths of each listener compared to the NH data at a group level of Klingel et al. (2021). Circles show 100-pps and triangles show 300-pps CI results. Squares show NH results. Pretest results are marked by open and posttest results are marked by filled symbols. Error bars indicate the variation across azimuths (standard error of the mean), individually for CI listeners and pooled across listeners for the NH data

Figure 7 shows the ITD weights of the individual listeners for further illustration. Overall ITD weights differed widely among listeners, ranging from essentially zero at both pulse rates in CI117 to about 0.5 for 100-pps stimuli in CI62. For five out of six listeners, the 100-pps posttest data point lies above the 100-pps pretest data point. The difference in ITD weighs between 100 and 300 pps was most pronounced for CI3 and CI62 and barely observable for CI117 and CI66 (the latter likely due to floor effects). For cross-study comparison, the NH ITD weights from Klingel et al. (2021) are shown on the far right. Subjecting the 100-pps CI results and the NH results (both averaged across azimuths) to a 2 (CI vs. NH) × 2 (pre- vs. posttest) mixed-design ANOVA yielded a significant effect of group (F(1,14) = 48.86, p < 0.001, η_p_^2^ = 0.777) and testing time (F(1,14) = 7.66, p = 0.015, η_p_^2^ = 0.354), but no significant testing time × group interaction (F(1,14) = 1.73, p = 0.210, η_p_^2^ = 0.110). This suggests overall stronger ITD weighting in NH listeners, but it provides no evidence for different amounts of reweighting in the two listener groups.

To test which listeners significantly changed the binaural-cue weighting for 100-pps stimuli, we used a metric that provides sufficient power for within-listener statistics, namely the ITD bias (in degrees) for each item. The ITD bias is defined as the difference between response and ITD azimuth in the direction of the ILD azimuth (i.e., a small bias indicates a large ITD weight, as the response is close to the ITD azimuth). Consistent-cue items were excluded. As in the weight analyses, either test phases 1 and 2 or test phases 2 and 3 were averaged to yield the pre- and posttest. We then ran a 2 (pre- vs. posttest) × 4 (cue disparity) RM ANOVA for each listener, since the bias is expected to be larger the further ITD and ILD azimuths are apart. This was confirmed by a significant main effect of cue disparity for each of the six listeners (all p < 0.001). There further was a significant main effect of time for CI66 (F(1,101) = 6.29, p = 0.014, Benjamini-Hochberg-corrected p = 0.036, η_p_^2^ = 0.059) with smaller ITD biases in the post- compared to the pretest, providing conservative within-listener statistical evidence that one of the six listeners significantly increased their ITD weighting for 100-pps stimuli. For CI3 (F(1,101) = 5.25, p = 0.024, Benjamini-Hochberg-corrected p = 0.052, η_p_^2^ = 0.049) and CI74 (F(1,101) = 5.10, p = 0.026, Benjamini-Hochberg-corrected p = 0.052, η_p_^2^ = 0.048), the main effect of time just missed the significance level after correcting for multiple comparisons.

### Discrimination Thresholds

**Fig. 8:**
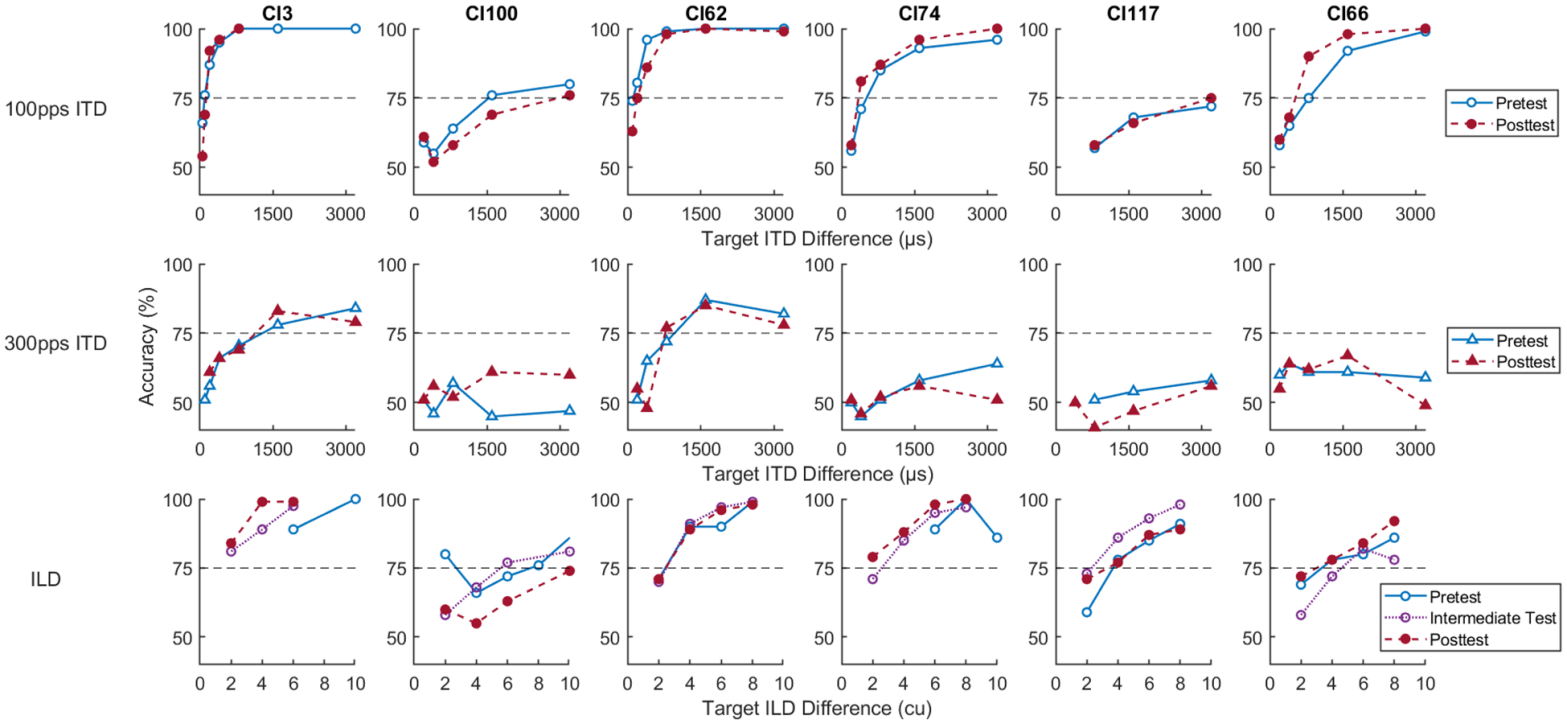
Psychometric functions of each listener (columns) for 100-pps ITDs (top row), 300-pps ITDs (middle row), and ILDs (bottom row). Since ILD psychometric functions were measured during all three test phases, “pretest” refers to test phase 1, “intermediate test” refers to test phase 2, and “posttest” refers to test phase 3. The x-axis shows the target ITD or ILD difference, i.e., two times the presented binaural cue

Figure 8 summarizes the results of the ITD and ILD sensitivity measurements. The individual panels show the psychometric functions for left/right discrimination performance of each listener, based on ITDs for 100-pps (top row) and 300-pps (middle row), and on ILDs for 100-pps (bottom row) pulse trains. The results for different test phases are indicated with different colors.

100-pps ITD thresholds were determinable for all listeners. For 300 pps, four out of the six listeners did not reach the targeted 75% performance level even for the largest ITD tested. For the other two listeners, thresholds were determinable, but much higher than at 100 pps. These results are consistent with the typically observed rate limitation in ITD sensitivity. Because of the undeterminable thresholds for most listeners, the 300-pps data were excluded from the statistical analyses. For ILDs (100 pps), thresholds were determinable although one listener (CI13) was so sensitive that even for the smallest possible ILD of one current unit, the performance exceeded the threshold criterion. Table 2 summarizes the thresholds estimated from the psychometric functions for the different conditions and test phases. Overall, performance varied widely among listeners for both ITD and ILD thresholds.

**Table 2:** ITD and ILD thresholds of the individual listeners. Denoted values correspond to two times the estimated thresholds. Since ILD thresholds were measured during all three test phases, “pretest” refers to test phase 1, “intermediate test” refers to test phase 2, and “posttest” refers to test phase 3

Listener	Pretest 100-pps target ITD difference (μs)	Posttest 100-pps target ITD difference (μs)	Pretest 300-pps target ITD difference (μs)	Posttest 300-pps target ITD difference (μs)	Pretest target ILD difference (cu)	Intermediate test target ILD difference (cu)	Posttest target ILD difference (cu)
CI3	96	118	1246	1260	5.08	2.32	1.72
CI62	134	202	782	784	2.96	2.78	2.86
CI66	682	460	N/A	N/A	4.70	5.64	4.52
CI74	500	390	N/A	N/A	5.76	3.12	2.44
CI100	1896	3006	N/A	N/A	5.98	7.40	10.42
CI117	3696	3174	N/A	N/A	4.62	3.06	4.18

The median estimated pre- and posttest ITD thresholds at 100 pps were 591 µs (IQR = 1762) and 426 µs (IQR = 2804), respectively. The thresholds were subjected to a Wilcoxon signed-rank test, which did not yield a significant effect (Z =  − 0.31, p = 0.753, r =  − 0.128), suggesting that the lateralization training had no effect on 100-pps ITD thresholds. ILD thresholds were subjected to a RM ANOVA with the factor testing time (test 1 vs. test 2 vs. test 3), as they were measured during all three test phases. Median thresholds were 4.89 cu (IQR = 1.13) for test 1, 3.09 cu (IQR = 2.87) for test 2 and 3.53 cu (IQR = 2.06) for test 3. The ANOVA did not yield a significant effect of testing time (F(2,10) = 0.42, p = 0.666, η_p_^2^ = 0.078), suggesting that the lateralization training had no effect on ILD thresholds.

### Relationship Between Cue Weights and Discrimination Thresholds

To determine if there is a relationship between the cue weights estimated from the lateralization task and the cue discrimination thresholds, we performed correlation analyses based on ITD weights and thresholds. Since ITD thresholds were unmeasurable for 300-pps pulse trains, we restricted these analyses to the 100-pps rate. For the ITD weights, the most central azimuths (at ± 3°) were selected because the ITD thresholds were also estimated at the perceived center (i.e., the reference stimulus had a zero ITD). Pretest ITD thresholds were not correlated with the pretest ITD weights (r(4) =  − 0.354, p = 0.491), suggesting that baseline ITD thresholds do not predict baseline ITD weighting.

### Response Compression

**Fig. 9:**
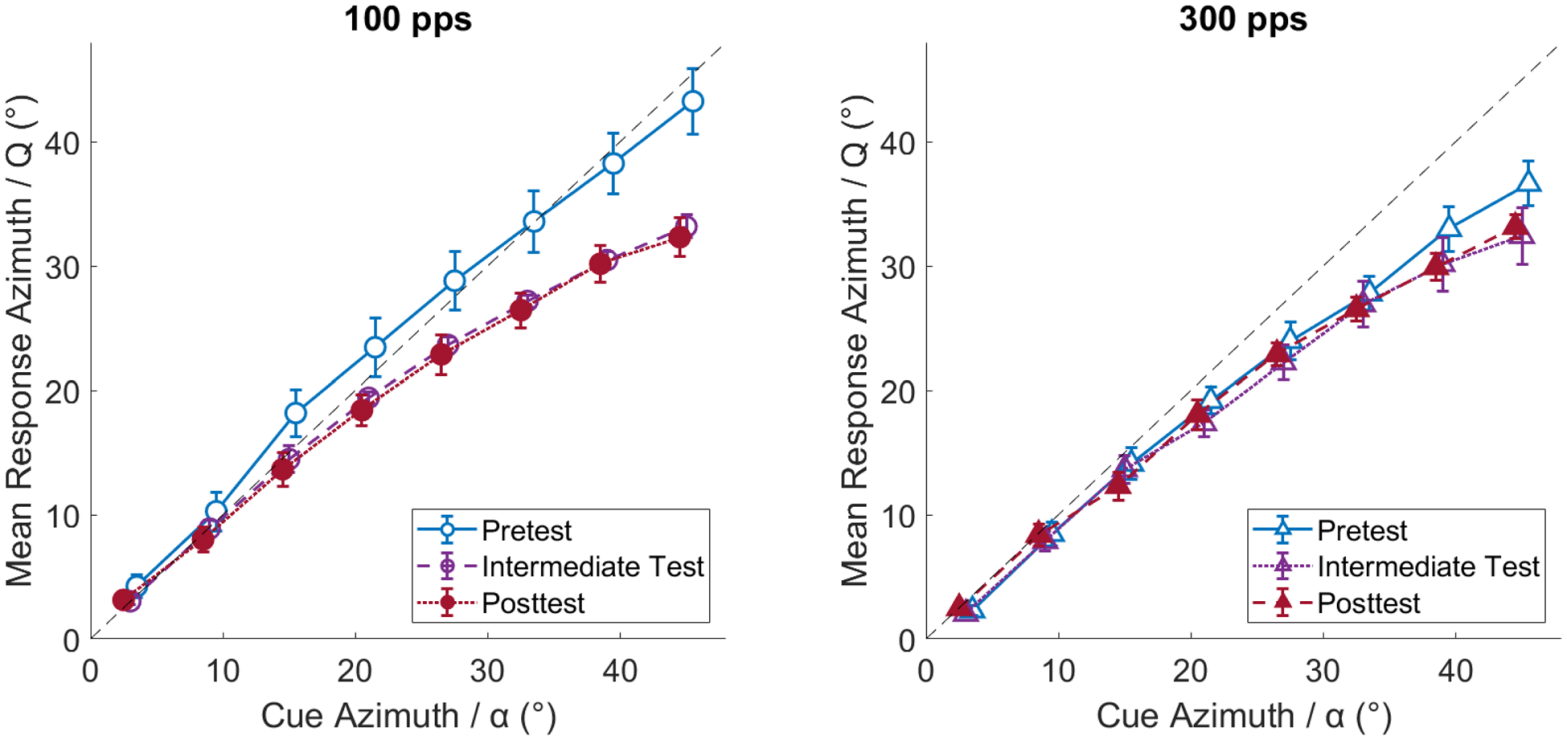
Mean response azimuths estimated by the factor Q of the regression analysis at each azimuth α. The left panel shows results for the 100-pps stimuli and the right panel shows the results for the 300-pps stimuli. “Pretest” refers to test phase 1, “intermediate test” refers to test phase 2, and “posttest” refers to test phase 3. Error bars show standard errors of the mean

In the previous NH study (Klingel et al. 2021), posttest responses were shifted towards the midline compared to pretest responses, which was attributed to the limited azimuth range of the reinforced cue compared to the perceived azimuth in the lateralization training. To evaluate this effect in the present study, Fig. 9 shows, separately for the three test phases, the response azimuth as a function of cue azimuth. The response azimuth was estimated by the parameter Q of the regression analysis, which considers all lateralization responses and is, therefore, a more reliable measure than only analyzing responses in consistent-cue conditions. For 100 pps, the slope of lateralization responses falls on the diagonal in test phase 1 and is shallower for test phases 2 and 3, which is consistent with Klingel et al. (2021). At 300 pps, the lateralization slope is already shallower in test phase 1 and differs only marginally thereafter. The shallower slope in test phase 1 for 300 compared to 100 pps is likely due to the unbalanced test order of the two rates: Since two listeners, who started with 100-pps training, were excluded from the study, most included listeners did the 300-pps test 1 after the 100-pps test 1; thus, after responses were already compressed. This conclusion is further supported by the observation that test phases 2 and 3 differed neither from each other nor across pulse rates (i.e., it cannot be explained by an effect of rate). Importantly, different lateralization slopes do not affect the binaural-cue weight estimates, because the overall lateralization slope affects parameters k_ITD_ and k_ILD_ equally and therefore cancels out in the final weight estimation (see Klingel et al. 2021).

### Response Variability

**Fig. 10:**
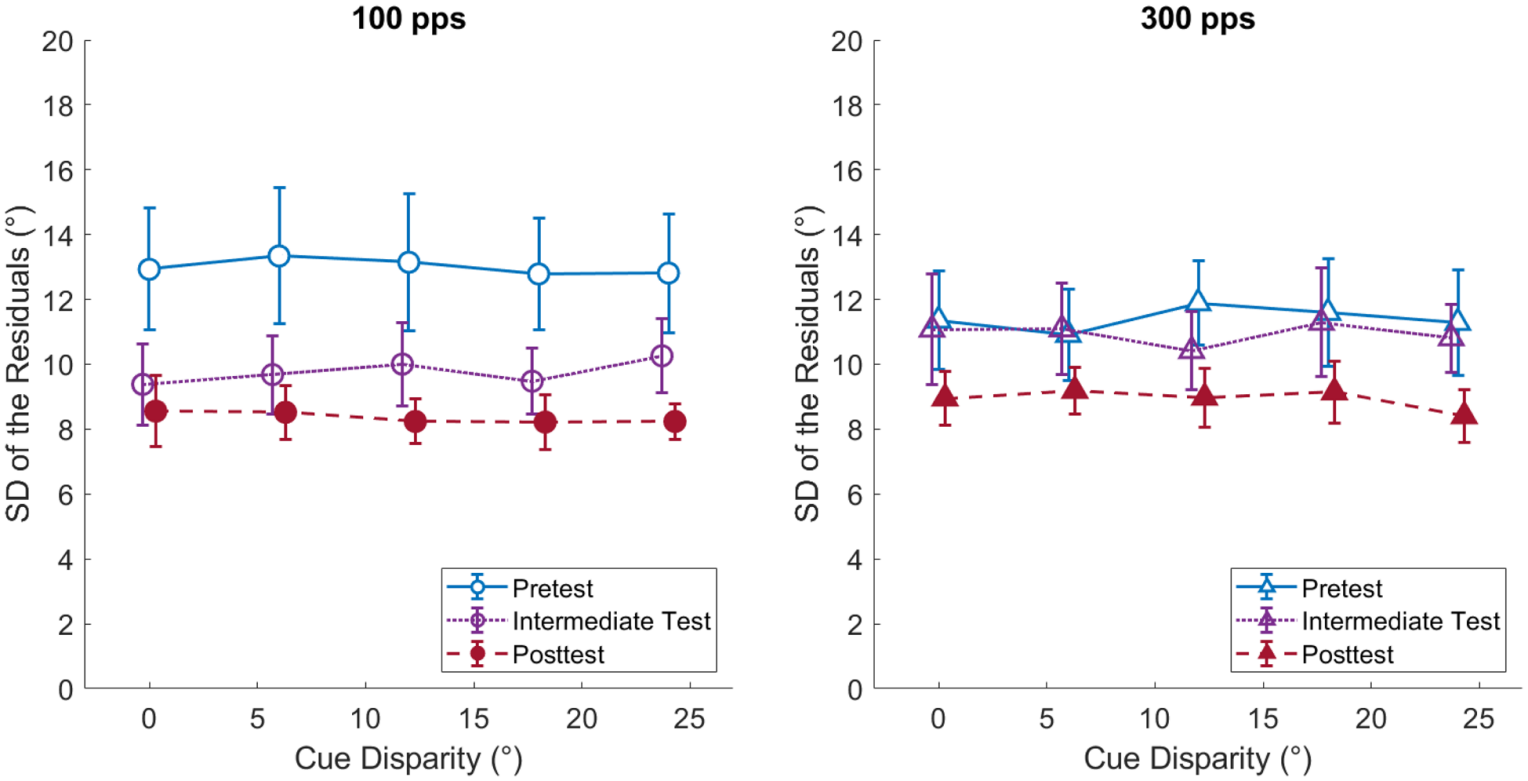
Mean response variability (defined here as the standard deviation of the residuals of the regression analysis) as a function of cue disparity in the three testing phases. The left panel shows results for the 100-pps stimuli, and the right panel shows the results for the 300-pps stimuli. “Pretest” refers to test phase 1, “intermediate test” refers to test phase 2, and “posttest” refers to test phase 3. Error bars show standard errors of the mean

The final examination of the impact of training concerned the lateralization precision, which refers to the consistency or variability in lateralization responses (see Heffner and Heffner 2005). To obtain a variability measure that is independent of binaural-cue weighting, the standard deviation of the residuals of the regression analysis (Eq. 2) was computed. The residual for a response is defined as the deviation of the actual response azimuth from the response azimuth predicted by the regression analysis. Figure 10 shows the response variability as a function of binaural cue disparity for the three testing phases (with the two pulse rates shown in separate panels). Averaged across pulse rates and cue disparities, lateralization precision improved from the first testing phase (12.20°) to the last testing phase (8.64°). For comparison, the NH study by Klingel et al. (2021) reported a similar relative improvement from their pretest (10.62°) to posttest (6.54°). The slightly higher absolute values are not surprising given that sound localization of CI listeners is overall worse than for NH listeners. This improvement in lateralization precision can be interpreted in terms of procedural training (i.e., by focusing attention or more precisely using the equipment) or overall perceptual training (Hawkey et al. 2004). Variability appears to be independent of cue disparity (i.e., the difference between the ITD and ILD azimuth of a stimulus), suggesting that listeners perceived a single, compact image for all cue disparities used. Finally, there is an apparent effect of pulse rate in test phase 1, showing higher variability for 100 pps than 300 pps, which can likely be attributed to the unbalanced test order, as already observed in the analysis of response compression: It is not surprising that response variability is higher in the very first lateralization test performed, which was the 100-pps condition in testing phase 1 for most listeners. This interpretation is consistent with the finding that such order effects did not influence testing phases 2 and 3. The above findings were supported by a 2 (100 vs. 300 pps) × 3 (test 1 vs. test 2 vs. test 3) × 5 (cue disparity) RM ANOVA, yielding a significant main effect of testing time (F(2,10) = 4.65, p = 0.037, η_p_^2^ = 0.482), no significant effect of cue disparity (F(4,20) = 0.14, p = 0.964, η_p_^2^ = 0.028), and a significant rate × testing time interaction (F(2,10) = 10.29, p = 0.004, η_p_^2^ = 0.673).

### Complementary Effects of the Two Binaural Cues

Apart from the effect of lateralization training, we were also interested in the practically relevant question if the addition of nonzero ITDs to nonzero ILDs significantly contributes to the extent of lateralization. For this purpose, we compared lateralization responses across an azimuth range of ± 27°, varying either ITDs (fixing ILDs roughly at zero), ILDs (fixing ITDs roughly at zero), or both ITDs and ILDs (i.e., presenting consistent-cue combinations). Since we did not test items with one of the cues set exactly to zero during the lateralization task, we approximated the zero position by averaging response azimuths for cue azimuths of − 3° and + 3°. Lateralization responses were averaged across all three test phases. Figure 5 shows the results for the pulse rates of 100 pps (left panel) and 300 pps (right panel). Linear-regression slopes fitted to the lateralization responses from Fig. 5 were significantly steeper for consistent cues than for fixed ITDs in both the 100-pps condition (T(5) = 2.24, p = 0.038, d_z_ = 0.92) and the 300-pps condition (T(5) = 3.64, p = 0.008, d_z_ = 1.49), suggesting that the addition of nonzero ITDs to nonzero ILDs significantly increases the extent of lateralization at both pulse rates. Finally, the slopes for fixed ILDs differed significantly from zero not only at 100 pps (M = 0.39, SD = 0.17, T(5) = 5.48, p = 0.001, d_z_ = 2.24) but also at 300 pps, even though the latter slope was close to zero (M = 0.11, SD = 0.12, T(5) = 2.25, p = 0.037, d_z_ = 0.92). This suggests that in conditions with fixed ILDs, location judgments were influenced by varying ITDs, but only marginally at 300 pps.

## DISCUSSION

We investigated a mechanism potentially contributing to the previous finding that sound localization with clinical CI systems is largely based on ILDs (Grantham et al. 2007; Seeber and Fastl 2008) and the extent of laterality evoked by ITDs is largely reduced in CI users compared to NH listeners, even when presented as well-controlled pulse-ITDs to the implanted electrodes via a research interface (e.g., Anderson et al. 2019; Laback et al. 2015). Specifically, we assumed that CI listeners reduce their ITD weighting based on their everyday experience with clinical CI systems, which lacks reliable and salient ITD cues while ILD cues are largely preserved. Therefore, we addressed the question if lateralization training can induce an increase in ITD weighting (i.e., reverse the maladaptive weighting) in CI listeners when pulse-ITDs are presented reliably and saliently via a research interface and are visually reinforced while ILDs are varied to render them unreliable.

A small but significant effect on a group level at 100 pps showed that the ITD weight can indeed be increased in CI listeners. On an individual level, one of the six tested listeners showed significantly increased ITD weights and two further listeners showed a strong trend for increased ITD weights. This suggests that, at least for some listeners, the low contribution of ITDs to lateralization in electric hearing is partially reversible with training once ITDs are presented saliently. Compared to NH data from Klingel et al. (2021), CI listeners’ ITD weighting was lower, consistent with the reduced ITD sensitivity in CI listeners and the assumption that CI listeners increase their ILD weighting based on the experience with their clinical devices. However, there was no evidence for different amounts of reweighting in the two listener groups.

In contrast, the 300-pps results showed no reweighting and ITD weights were consistently lower compared to 100 pps. Together with the observation that ITD thresholds for 300-pps stimuli exceeded the range of tested ITDs (100–1600 μs) for four of our six listeners, this suggests that the mechanism causing the rate limitation for ITDs is independent of the mechanism responsible for binaural-cue reweighting. Rather, this pattern is consistent with data from NH listeners presented with envelope-ITD cues: Stecker (2010) observed that ITD/ILD trading ratios increasingly favored ILDs over ITDs as the envelope rate of Gabor click trains (centered at 4 kHz) increased, as predicted based on the established rate limitation for envelope-ITDs. Thus, the results support the interpretation that (a) pulse-ITDs in electric pulse trains and envelope-ITDs in acoustic hearing provide comparable information to the binaural processing stages, and (b) the upper rate limit of that binaural pathway prevents reweighting for 300-pps electric pulse trains.

As binaural-cue weights do not change without intervention in NH listeners (Klingel et al. 2020), it was considered unlikely that simple re-testing would lead to reweighting in CI listeners. Such an explanation for the 100-pps results is also ruled out by the null effect at 300 pps, which thus serves as a control condition. The null effect at 300 pps further suggests that onset ITD cues did not play a major role in the present results, as they were equally present in the 100- and the 300-pps conditions.

In addition to binaural-cue weights, binaural-cue thresholds were measured to test whether the lateralization training influences cue sensitivity. Neither ITD nor ILD thresholds systematically differed between the test phases, suggesting that the training had no influence on binaural-cue sensitivity. The range of ITD thresholds we measured is consistent with previous studies (see Laback et al. 2015). ILD sensitivity was very good overall, consistent with previous studies using either direct stimulation (e.g., Lawson et al. 1998) or clinical processors (e.g., Laback et al. 2004). For four out of six listeners, the estimated ILD thresholds approached or even fell below the smallest current level difference realizable with the implants.

We were further interested in whether binaural-cue weights and binaural-cue thresholds are related. Interestingly, ITD weights did not correlate significantly with ITD thresholds. This suggests that the weight and threshold estimates are independent from each other. The lack of a significant correlation between these measures could be due to the small number of listeners and, thus, insufficient statistical power. However, it might also be due to differences between the two tasks: While binaural-cue thresholds were measured with a relative task as listeners had to detect shifts relative to a reference stimulus, binaural-cue weights were measured with an absolute task as listeners had to indicate the perceived azimuth of the stimulus. Furthermore, thresholds were estimated at the center, while weights were estimated at different azimuths between ± 45°. We tried to address this by only considering the estimated weight at ± 3° azimuth (i.e., the most central azimuth tested during the lateralization task) for the correlation analysis, but since reweighting appears to be stronger at lateral azimuths, measuring thresholds at lateral azimuths might have been more informative. Finally, either ITDs or ILDs were set to zero during threshold estimation, while both ITDs and ILDs varied during the binaural-cue weight estimation, which thus may involve interactions between ITD and ILD cues. Therefore, it might be interesting for future studies to use not only threshold measurements but also tasks involving supra-threshold cue combinations (as in the present binaural-cue weight measurement) to predict performance in real-life situations where the two binaural cues interact.

In the present study, the estimated ITD weights were overall smaller at more lateral azimuths compared to central azimuths. A similar pattern was observed in NH listeners (Klingel et al. 2020, but also see Klingel et al. 2021). The current results might, however, also be attributable to an insufficient perceptual match in linking ITDs and ILDs to azimuths. While we selected the ILD values on an individual basis during the parameter determination to encompass the targeted azimuthal range, we used a fixed range of ITD cues, which was only crudely adapted to the individual listener’s sensitivity. While this limits the interpretability of the effect of azimuth, it should be noted that our primary interest was the overall effect of lateralization training on cue weighting and different azimuths were tested primarily for explorative purposes and to avoid that listeners respond strategically (i.e., memorizing specific cue-combinations and locations).

The neural mechanisms underlying binaural-cue reweighting are still unknown. A recent study demonstrated improved neural ITD sensitivity in the inferior colliculus of early-deafened rabbits that were chronically exposed to ITD cues consistent with the visual environment during their developmental period as compared to early-deafened rabbits that were not exposed to ITD cues (Sunwoo et al. 2021). This effect was found even at high rates (around 300 pps) for which we did not observe reweighting. However, besides the difference in species, a potentially important difference between their and our study is that our human listeners experienced deprivation from binaural cues and completed our lateralization training during adulthood, where plastic changes may occur at different neural sites (but see Tirko and Ryugo 2012). Future physiological studies may address the reweighting mechanism in adulthood.

Finally, unrelated to binaural-cue reweighting, we took the opportunity to address the practically relevant question if the addition of nonzero ITDs to nonzero ILDs significantly contributes to the extent of lateralization. Presenting spatially consistent ILD and ITD cues indeed produced steeper functions of lateralization responses than presenting stimuli with ITDs fixed at roughly zero not only for 100-pps but also for 300-pps stimuli. This complementary effect of providing spatially consistent ILD and ITD cues is remarkable particularly for the 300-pps rate, for which presenting ITDs alone (i.e., fixing ILDs at roughly zero) caused rather shallow functions (though differing significantly from zero) and ITD thresholds were extremely high or undeterminable. This suggests that providing reliable pulse-ITD cues with future stimulation strategies might aid lateralization even for stimulation rates for which ITD-based measures reveal little to no evidence for sensitivity.

A general limitation of the present study is the small number of six bilateral implantees that could be recruited to complete this time-consuming study. Also, because only one of the six listeners showed significant reweighting at 100 pps based on conservative within-participant analyses, the current results do not allow generalization to the population of bilateral implantees, which is known to be heterogeneous. Future studies should, thus, extend the pool of participants and identify factors responsible for the variability in outcomes.

Another potential limitation is the unbalanced number of listeners who started training with either pulse rate in our experiment, which resulted from the need to exclude two listeners included in the original design. If reweighting would generalize across pulse rates, this might have favored reweighting for 100-pps stimuli as the total training time was longer after completing both lateralization training phases. However, we believe that this would be accounted for by averaging across test phases: Since testing phases 1 and 2 were averaged to constitute the 100-pps pretest for these listeners, a potential reweighting effect induced by the 300-pps training already influences the 100-pps pretest, thus reducing the pre vs. post difference. Another potential source of asymmetry is that the averaging of test phases leads to more accurate estimates for 100-pps pretests and 300-pps posttests than for 100-pps posttests and 300-pps pretests for most of the listeners. However, there is no reason to assume that this would systematically affect the pre vs. post difference. In summary, we believe that the unbalanced test order did not systematically affect the reweighting results reported here.

The present results are directly applicable for stimulation strategies that aim to encode ITD cues via the pulse timing using low stimulation rates. To extend the findings towards envelope-coding strategies, future studies may investigate, whether ITD weighting can also be increased for high-rate pulse trains conveying enhanced envelope-ITD cues either via envelope-shape modification (Monaghan and Seeber 2016; Francart et al. 2014) or via short interpulse-interval pulses inserted at a low rate (Srinivasan et al. 2018). Because both low-rate pulse trains and high-rate pulse trains with low-rate modulation likely activate the same binaural pathway (Srinivasan et al. 2020; van Hoesel et al. 2009), the current findings will likely extrapolate to the latter stimulus types. Since ITDs may be particularly important for azimuth ranges where ILDs change non-monotonically with azimuth, in acoustic situations where ILDs are not robust (Macaulay et al. 2010), and for understanding speech in multi-talker environments (e.g., Kidd et al. 2010), increased ITD weighting could be particularly helpful in these situations.

In conclusion, by reinforcing ITD cues during a lateralization training, listeners significantly increased the perceptual weight given to pulse-ITDs conveyed by low-rate pulse trains (100 pps). For higher-rate pulse trains (300 pps), however, no reweighting was found, suggesting that separate mechanisms underlie the well-known increase of ITD thresholds with increasing pulse rate and binaural-cue reweighting. Apart from this rate dependency, binaural-cue weights and binaural-cue thresholds were not significantly correlated. The present results are promising in terms of making low-rate ITD cues better usable for sound localization with future CI systems that convey reliable and salient ITD information.

## Footnotes

1 Because ITDs exceeding about one-third of the inter-pulse interval can potentially introduce a left/right ambiguity in the ongoing signal, we checked the potential influence of such an effect. Removing possibly “critical” conditions (i.e., an alpha of 45° for the 300-pps condition) from the weight analysis did not impact the outcome.

2 A 2 (100 vs. 300 pps) × 10 (azimuth) ANOVA yields neither a significant effect of rate (*F*(1,5) = 0.32; *p* = .595; *η*_*p*_^*2*^ = .060) nor a significant rate × azimuth interaction (*F*(9,45) = 0.80; *p* = 616; *η*_*p*_^*2*^ = .138).

3 Before mirroring the data, we tested whether the ITD weights at the two hemispheres differed significantly. This was not the case (*F*(1,5) = 0.06; *p* = .818; *η*_*p*_^*2*^ = .012).

4 Here and in the remainder of the paragraph, the “other” (counterpart) cue is referred to in parentheses.
